# Tyrosine 625 plays a key role and cooperates with tyrosine 630 in MPL W515L-induced signaling and myeloproliferative neoplasms

**DOI:** 10.1186/s13578-016-0097-3

**Published:** 2016-05-23

**Authors:** Chunjie Yu, Qiong Yang, Yuhong Chen, Demin Wang, Ross Levine, John Crispino, Qiang Wen, Zan Huang

**Affiliations:** College of Life Sciences, Wuhan University, 16 Luo-Jia-Shan Road, Wuhan, 430072 Hubei People’s Republic of China; Feinberg School of Medicine, Department of Medicine, Division of Hematology and Oncology, Northwestern University, 303 E Superior Street, Lurie Research Building 5-250D, Chicago, IL 60611 USA; Blood Research Institute, Blood Center of Wisconsin, Milwaukee, WI 53226 USA; Human Oncology Program and Pathogenesis Program and Leukemia Service, Department of Medicine, Memorial Sloan Kettering, New York, NY USA

**Keywords:** Myeloproliferative neoplasms (MPN), MPL W515L, MPL515/625/630, STAT5, AKT, In vivo

## Abstract

**Background:**

Myeloproliferative neoplasms (MPN) are a group of blood cancers that boost normal blood cell production in the bone marrow. Abnormal mutations in stem cells were found accompanying with the occurrence of MPN. It has been shown that MPL mutations (MPL W515L or MPL W515K) were involved in patients with MPN. Since tyrosine residues 625 and 630 mediate normal MPL signaling, whether them affect MPL W515L-induced myeloproliferative neoplasms (MPNs) is unknown.

**Results:**

In this study, we further tested their functions in MPL W515L-induced myeloproliferative neoplasms (MPNs) by substituting either or both of them with phenylalanine in MPL W515L (termed as MPL515/625, MPL515/630 and MPL515/625/630, respectively). In vitro, MPL515/630 but not MPL515/625 or MPL515/625/630 retained the ability to induce TPO-independent proliferation and increase colony-forming unit megakaryocytes (CFU-Mk). Accordingly, differential activation of the downstream signaling by four mutants was observed and constitutively active STAT5 or AKT instead of STAT3 partially compensated MPL515/625/630 function. Further support this, STAT5-deficiency impaired MPL W515L-induced CFU-Mk expansion. In vivo, MPL515/630 but not MPL515/625 or MPL515/625/630 induced typical features of MPNs with high WBC and platelet counts, splenomegaly, hepatomegaly and hypercellularity in the bone marrow. Surprisingly, MPL515/625 also caused hypercellularity of bone marrow and splenomegaly without any other significant features. We also observed differential effects of the four mutants on progenitors, myeloid cells and megakaryocytes.

**Conclusions:**

Our studies have revealed distinct features of tyrosine sites 625 and 630 in mediating MPL W515L-induced megakaryocyte hyperproliferation and MPNs. Our study also suggests that MPL cytosolic phosphorylated Y625 and flanking amino acids could become targets for pharmacologic inhibition in MPNs.

**Electronic supplementary material:**

The online version of this article (doi:10.1186/s13578-016-0097-3) contains supplementary material, which is available to authorized users.

## Background

*JAK2* and *MPL* mutations are associated with BCR-ABL negative myeloproliferative neoplasms (MPNs) including polycythemia vera (PV), essential thrombocythemia (ET), and primary myelofibrosis (PMF) [1–3]. These mutations caused activation of JAK2, cytokine independent growth of human blood cells, and induced MPNs in mouse models [4–7]. Although Ruxolitinib the only FDA approved JAK2 inhibitor alleviates symptoms of PMF, it does not decrease the mutant allele burden [8, 9]. Besides developing new types of JAK2 inhibitor, other downstream signaling pathways activated by these mutants were also proposed to be potential targets for MPNs therapy.

*JAK2* and *MPL* mutations primarily activate JAK/STAT, PI3K/AKT and MAPK/ERK signaling pathways. JAK/STAT pathway was the most significant signature observed in MPNs even in patients without the *JAK2* or *MPL* mutation [10]. Indeed, STAT5 was required for BCR-ABL or JAK2-induced MPNs [11] and constitutively active STAT5 caused MPNs [6, 12, 13]. STAT3 supported K-Ras G12D-induced MPNs [14]. Besides JAK/STAT pathway, activation of PI3K/AKT is another obvious consequence of *JAK2* and *MPL* mutations. PI3K mutations have been identified in some types of cancers but not leukemia [15–17]. However, constitutively active AKT caused leukemia and lymphoma in mouse suggesting a potential role of PI3K/AKT in hematological malignancy [18]. Recently, PI3K/AKT inhibitor was shown to reduce disease burden in MPNs mouse model and the combinatory application of JAK2 inhibitor and PI3K/AKT inhibitor had synergistic effect on causing apoptosis in leukemic cells with JAK2 V617F, suggesting PI3K/AKT as a potent target for MPNs therapy [19–21]. Contrast to PI3K/AKT pathway, the role of MAPK/ERK activation in MPNs was elusive. In an *Nf1*-null mouse model, MAPK/ERK was required for MPNs development [22]. In a bone marrow transplantation model, constitutively active MEK caused MPNs [23]. However, its role in typical MPNs with *JAK2* or *MPL* mutations had not been addressed.

In this study, we set out to evaluate the role of downstream signaling pathways in MPL W515L-induced signaling and MPNs. Based on previous knowledge of tyrosine 625 and 630 (also known as Y112 and Y117 numbering corresponds to cytosolic residues in mouse MPL) in normal MPL signaling, we expected to manipulate MPL W515L-induced downstream signaling by substituting these two sites [24, 25].

## Methods

### Plasmid construction and gene expression

Human MPL cDNA (NCBI Reference Sequence: NM_005373.2) was subcloned into XhoI site of the pMSCV puro retroviral vector and used as a template for MPL Y625F or Y630F mutations known as Y112 and Y117 numbering corresponds to cytosolic residues in mouse MPL [25]. G1ME cells overexpressing MPL 515/625/630 were further transduced with retroviruses encoding STAT5A 1*6, STAT3C, AKT1 CA, NRASD12, or vector control (pMIGR1). Forty-eight hours after transduction, cells were washed twice and cultured with or without TPO for indicated time. The percentage of GFP-positive cells was analyzed by flow cytometry.

### Western blot analysis

Western blot analysis was performed as previously described [26]. Antibodies used in this study included mouse anti-HSC70 (Santa Cruz Biotechnology, Santa Cruz, CA, USA); rabbit anti-Shc, anti-phospho-Shc, anti-Stat5, anti-phospho-Stat5, anti- Stat3, anti-phospho-Stat3, anti-AKT, anti-phospho-AKT, anti-phospho-ERK, anti-phospho-ERK, (Cell Signaling Technology, Beverly, MA, USA). HSC70 antibodies served as loading controls.

### Animals and megakaryocyte cultures

Wild-type C57BL/6 were purchased from Jackson. Mice with one copy of floxed *Stat5a* and *Stat5b* alleles, one copy of deleted *Stat5a* and *Stat5b* alleles, and Mx1-Cre transgene were indicated as F/−. Mice with one copy of floxed *Stat5a* and *Stat5b* genes, one copy of intact *Stat5a* and *Stat5b* alleles, and Mx1-Cre transgene were indicated as F/+. Mice with two copies of floxed *Stat3* genes and Mx1-Cre transgene were indicated as STAT3F/F. All mouse strains were maintained in microisolator housing within a barrier facility. Animal studies were approved by the Northwestern University and Wuhan University Animal Care and Use Committees. Primary megakaryocyte liquid culture was performed as previously described [26]. G1ME cells were maintained in a minimal essential medium supplemented with 20 % fetal bovine serum and 1 % thrombopoietin (TPO) conditional medium [26]. The inhibitors used to treat G1ME/MPL W515L cells were JAK2 Inhibitor II (50 μM, Cat#1837-91-8, Calbiochem), LY29402 (20 μM, Cat#154447-36-6, Calbiochem), BEZ235 (10 μM, Cat#915019-65-7, SIGMA-ALDRICH), MK2206 (2 μM) provided by Merck (Rahway, NJ, USA), AKT Inhibitor V (10 μM, Cat#35943-35-2, Calbiochem), AKT Inhibitor IV (10 μM, Cat#681281-88-9, Calbiochem), AKT Inhibitor VIII (10 μM, Cat#612847-09-3, Calbiochem), Rapamycin (5 μM, Cat#53123-88-9, SIGMA-ALDRICH), GF109203X (5 μM, Cat#133052-90-1, SIGMA-ALDRICH), Salirasib (50 μM, Cat# 162520-00-5, SIGMA-ALDRICH), and PD98059 (20 μM, Cat#167869-21-8, SIGMA-ALDRICH).

### Colony-forming unit assay

Mouse bone marrow progenitor cells were enriched by using a mouse hematopoietic progenitor cell enrichment kit (Cat# 19756, Stemcell Technologies, Vancouver, BC, Canada) and cultured in expansion medium (RPMI 1640 medium supplemented with 10 % FBS, 10 ng/ml IL-3, 10 ng/ml IL-6, 20 ng/ml SCF) overnight. Cells were further transduced with retrovirus expressing genes of interest as previously described [26]. The infected cells were further selected with puromycin (1 μg/ml) for 24 h. 2 × 10^3^ or 2 × 10^4^ cells were used for colony forming unit-myeloid cells (CFU-Myeloid) or megakaryocytes (CFU-Mk) as previously described [27].

### Bone marrow transplantation

Bone marrow transplantation was performed as previously described [28]. Briefly, bone marrow progenitor cells were transduced with a retrovirus overexpressing MPL mutants and GFP. The transduced cells were transplanted into lethally (1100 rad) irradiated syngeneic recipient mice. The disease burden in the recipient mice was analyzed 1 month after transplantation.

### Flow cytometry

Megakaryopoiesis in vitro was analyzed by staining cells with anti-CD41 or anti-CD42 antibodies and 4′, 6-diamidinio-2-phenylindole (DAPI) as previously described [26]. A gate was set for CD41^+^ cells for analysis of polyploidy. Analysis of HSC, HPC, GMP, CMP and MEP in the bone marrow was performed as previously described [1–3]. To analyze the erythrocytes, myeloid cell, and megakaryocytes in the bone marrow, antibodies against Ter119-APC, CD41-PEcy7, Gr-1-APC and Mac-1-V450 were used.

### Statistics

All data were presented as Mean ± SD. Statistical analyses were performed using the Student’s *t* test (two tailed, unpaired). A *p* value of 0.05 or less was considered as significance.

## Results

### Tyrosine 625 plays a critical role in MPL W515L-induced hyperproliferation of megakaryocytes

**Fig. 1:**
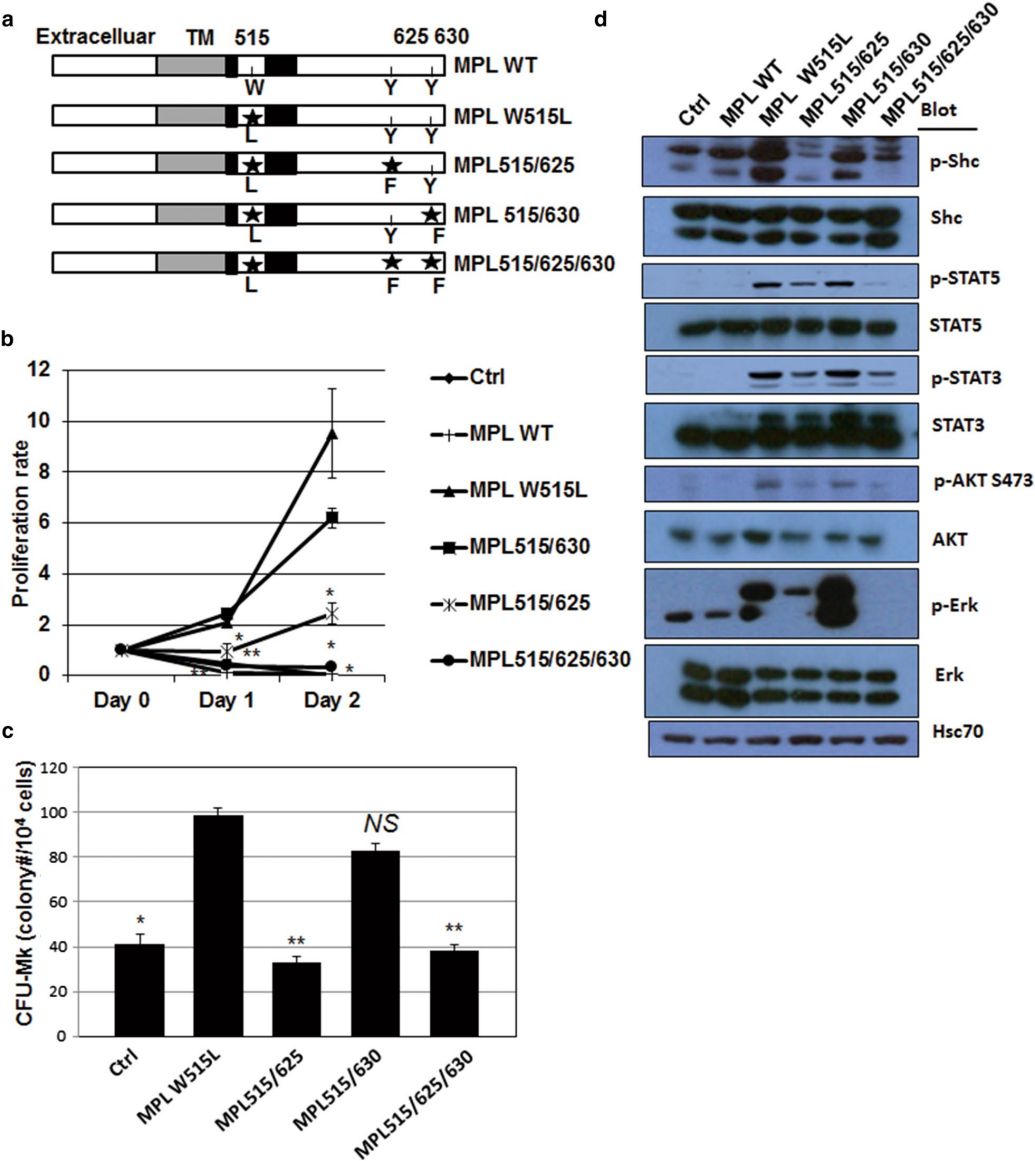
Tyrosine 625 is critical for MPL W515L-induced TPO-independent cell proliferation and megakaryocyte hyperproliferation. **a** Diagram illustrates the wild-type MPL and four MPL mutants. *Stars* and *numbers* indicate mutation sites. **b** G1ME cells transduced with each construct were cultured without TPO. The cell numbers were counted over time and normalized to the starting cell numbers (Day 0) as proliferation rate. **c** WT bone marrow progenitor cells transduced with each construct were used for CFU-Mk assay. The *bar graphs* are the statistics of one representative experiment with duplicates from two independent experiments with similar results. *Asterisk* indicates significance (*p* < *0.05*) and *NS* indicates non-significance compared to MPL W515L. **d** Cells were cultured in the absence of TPO and harvested for western blot to detect the phosphorylation and the expression level of each protein as indicated. Hsc70 is the loading control

Previously, we observed that overexpression of JAK2 V617F or MPL W515L conferred TPO-independent growth in G1ME cells [26]. In fact, MPL W515L potently increased colony-forming unit-myeloid cells (CFU-Myeloid) and colony-forming unit-megakaryocytes (CFU-Mk) that led to decreased polyploidy in mature megakaryocytes (Additional file 1: Figure S1a–c). In vivo, MPL W515L induced leukocytosis and thrombocytosis (Additional file 1: Figure S1d). Noticeably, MPL W515L caused phosphorylation of STAT3, STAT5, AKT, ERK and JNK (Additional file 1: Figure S1e). Tyrosine sites 625 and 630 are known to mediate normal MPL signaling by docking STAT3 and STAT5 [25, 29]. However, whether MPL W515L requires these tyrosine sites has not been addressed. To test this, we substituted tyrosine 625 and/or tyrosine 630 with phenylalanine on MPL W515L and termed these mutants as MPL515/625 (MPL W515L with further Y625 substituted by phenylalanine), MPL515/630 (MPL W515L with Y630 substituted by phenylalanine), and MPL515/625/630 (MPL W515L with both Y625 and Y630 substituted by phenylalanine), respectively (Fig. 1a). In G1ME cells, a TPO-dependent GATA-1-null mouse megakaryocyte cell line, all constructs did not significantly affect cell proliferation in the presence of TPO (Additional file 1: Figure S2a). Upon TPO withdrawal, MPL W515L conferred the cells of TPO-independent proliferation while control vector (Ctrl) or MPL WT did not (Fig. 1b). Interestingly, MPL515/625 showed significantly impaired ability to do so while the ability of MPL515/630 was only modestly affected (Fig. 1b). In contrast, MPL515/625/630 completely lost its ability (Fig. 1b). Consistent to these observations, MPL W515L and MPL515/630 induced dramatic increase of CFU-Mk in mouse bone marrow progenitor cells while MPL515/625 and MPL515/625/630 did not (Fig. 1c). These findings suggest that tyrosine 625 plays a critical role and may cooperates with tyrosine 630 in MPL W515L-induced megakaryocyte hyperproliferation.

To examine the effect of tyrosine 625 and/or 630 on the downstream signaling, we measured the phosphorylation of STAT3/5, AKT, Erk and Shc. In the presence of TPO, none of constructs significantly altered the phosphorylation status (Additional file 1: Figure S2b). Upon TPO withdrawal, MPL W515L maintained phosphorylation of all five molecules but control vector (Ctrl) or MPL WT did not (Fig. 1d). Moreover, MPL515/625 showed impaired ability to do so (Fig. 1d). In contrast, MPL515/630 retained the ability comparable to MPL W515L (Fig. 1d). Of note, MPL515/625/630 further decreased the phosphorylation of STAT5 and Erk but not other three molecules compared to MPL515/625 (Fig. 1d). Considering the difference of MPL515/625 and MPL515/625/630 in inducing cell proliferation and STAT5 and Erk phosphorylation, STAT5 and Erk may be important in MPL W515L-induced hyperproliferation of megakaryocytes.

### Critical roles of STAT5 and AKT in megakaryocyte proliferation and compensating the loss function of MPL515/625/630

**Fig. 2:**
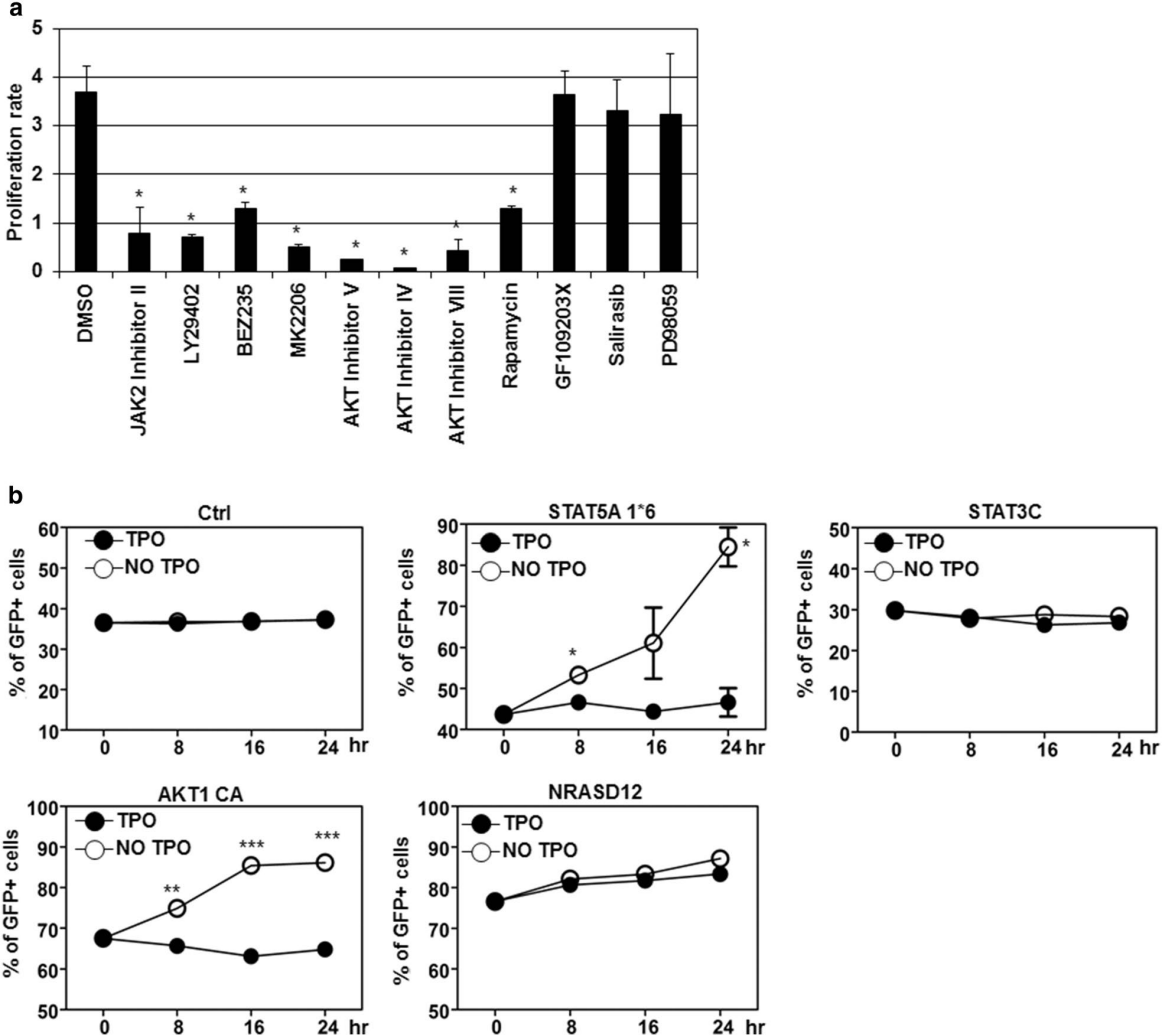
Critical roles of STAT5 and AKT in MPL W515L-induced hyperproliferation and compensating the loss function of MPL515/625/630. **a** MPL W515L- transduced G1ME cells were treated with DMSO or inhibitors as indicated. Cell numbers were counted and normalized to the starting cell numbers (Day 0) as proliferation rate. **b** MPL515/625/630-transduced G1ME cells were further infected with control GFP-expressing retrovirus or virus expressing GFP and proteins as indicated. The percentages of GFP^+^ in the resultant cells cultured with (*solid round*) or without TPO (*hollow round*) over time were analyzed by flow cytometry. *, **, *** indicates *p* < 0.05, 0.01, 0.001, respectively

We further dissected the role of each downstream signaling pathway in MPL W515L-induced cell proliferation by adding specific inhibitors to the cell culture. We chose appropriate doses that were previously shown to inhibit signaling efficiently [19, 30–32]. As a control, JAK2 inhibitor potently suppressed cell proliferation (Fig. 2a). Inhibitors including LY29402, BEZ235, MK2206, AKT inhibitor V, IV, VIII, and Rapamycin that were specific for PI3K/AKT pathway also efficiently repressed cell proliferation (Fig. 2a). In contrast, inhibitor for PKC (GF109203X) or MAPK/ERK (PD98059) had no significant effect (Fig. 2a). These results suggest an important role of PI3K/AKT pathway in MPL W515L-induced megakaryocyte hyperproliferation [19]. To further verify these results, we took advantage of MPL515/625/630 that failed to stimulate cell proliferation and all three signaling pathways (Fig. 1). We restored each signaling pathway by introducing constitutively active form of STAT3, STAT5, AKT1 or Ras into these cells. Overexpression of each of these molecules alone did not allow us to culture these cells for long term in the absence of TPO (data not shown). If any of these molecules contributed to MPL W515L-induced hyperproliferation or survival, we would expect to observe increase of the transduced cells by monitoring the bi-cistronically expressed GFP. As a control, GFP^+^ cells were not altered for control vector (Ctrl) under both culture conditions (Fig. 2b). Apparently, the GFP^+^ cells with constitutively active STAT5 (STAT5A 1*6) or AKT1 (AKT1 CA) increased over time in the absence of TPO but not significantly altered in the presence of TPO (Fig. 2b). In contrast, the GFP^+^ cells for constitutively active STAT3 (STAT3C) was not changed at all in both conditions similar to control vector (Fig. 2b). Consistent with inhibitor experiment, constitutively active Ras (NRASD12) did not significantly increase GFP^+^ cells in the absence of TPO compared to that in the presence of TPO [33, 34]. Thus we confirmed that STAT5 and AKT were two important factors contributing to MPL W515L-induced hyperproliferation or survival of megakaryocytes.

### STAT5-deficiency impairs MPL W515L-induced hyperproliferation of megakaryocytes

**Fig. 3:**
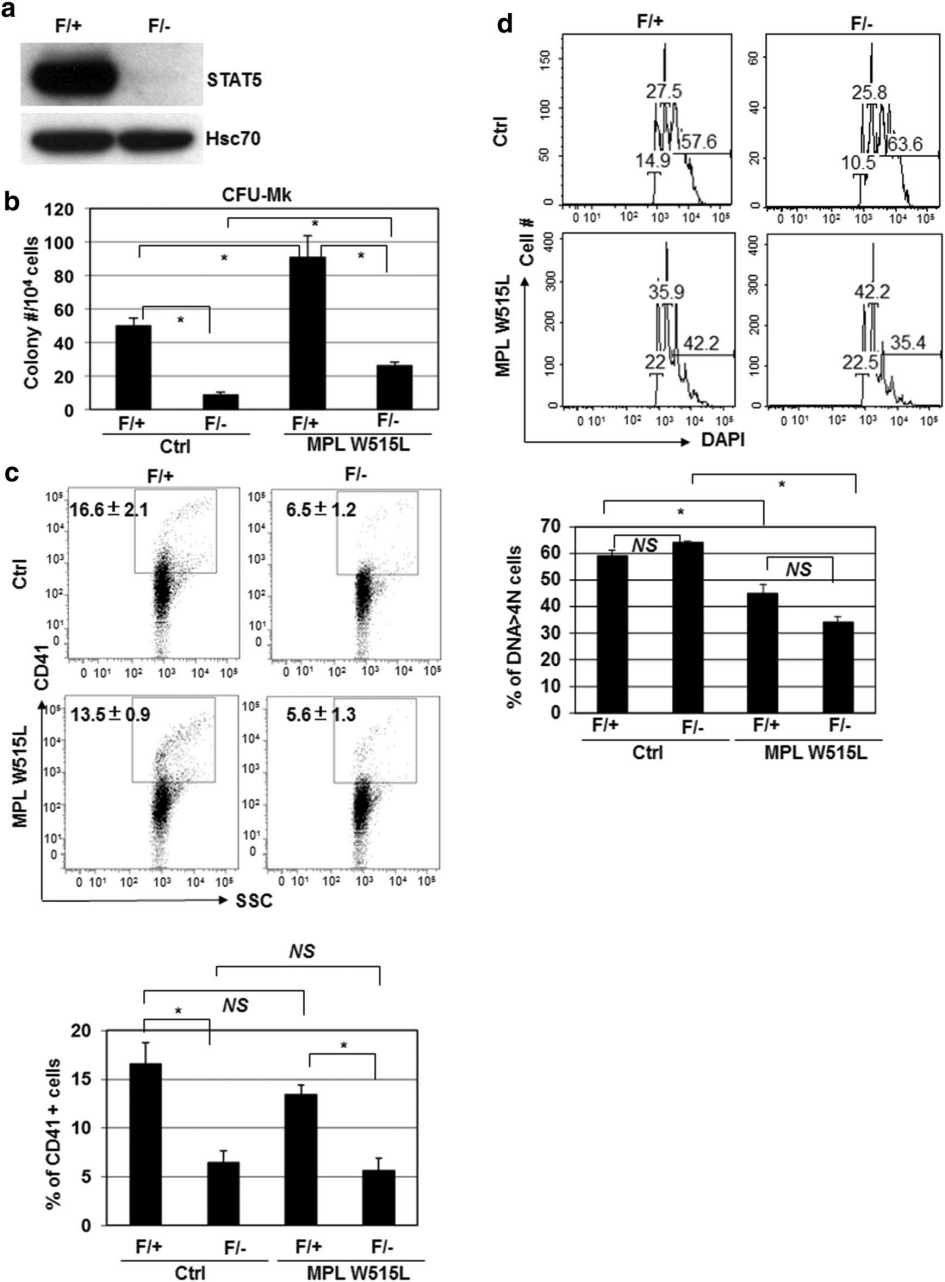
STAT5-deficiency impairs MPL W515L-induced megakaryocyte hyperproliferation. **a** Bone marrow cells from F/− or F/+ mice treated with poly I-C were used for western blot to detect the total STAT5 (STAT5A and STAT5B). Hsc70 was the loading control. **b** F/+ and F/− bone marrow progenitor cells were transduced with control retrovirus (Ctrl) or MPL W515L-expressing virus. The resultant cells were purified and used for CFU-Mk. **c** The transduced cells were also cultured for megakaryocyte differentiation to detect CD41 expression by flow cytometry. **d** The DNA content in CD41^+^ cells was also measured by staining cells with DAPI. *Numbers* indicate the percentage of gated cells. Cells with DNA > 4N were polyploid cells for statistics. All *bar graphs* are the statistics of one representative experiment (duplicate) from two independent experiments with similar results. *Asterisk* indicates significance (*p* < *0.05*) and *NS* indicates non-significance

STAT5 is required for BCR-ABL to induced CML in a mouse model [35]. However, its role in MPL W515L-induced hyperproliferation of megakaryocytes has not been fully addressed. To further study this, we overexpressed MPL W515L in STAT5-deicient (F/−) or littermate control (F/+) bone marrow cells (Fig. 3a). In control F/+ cells, MPL W515L overexpression significantly expanded CFU-Mk and reduced polyploidy without altering CD41 expression (Fig. 3b–d). STAT5 deficiency (F/−) reduced CD41 expression without altering the polyploidy compared to littermate control even in the presence of MPLW515L, suggesting that STAT5 is essential for megakaryopoiesis (Fig. 3c, d). Notably, STAT5-deficiency significantly impaired MPL W515L-induced increase of CFU-Mk compared to littermate control cells, suggesting an important role of STAT5 in MPL W515L-induced hyperproliferation of megakaryocytes. Interestingly, the number of CFU-Mk induced by MPL W515L in STAT5-deficient mouse bone morrow cells were about threefold higher than their corresponding vector control, suggesting a STAT5-independent mechanism by which MPL W515L causes hyperproliferation of megakaryocytes.

**Fig. 4:**
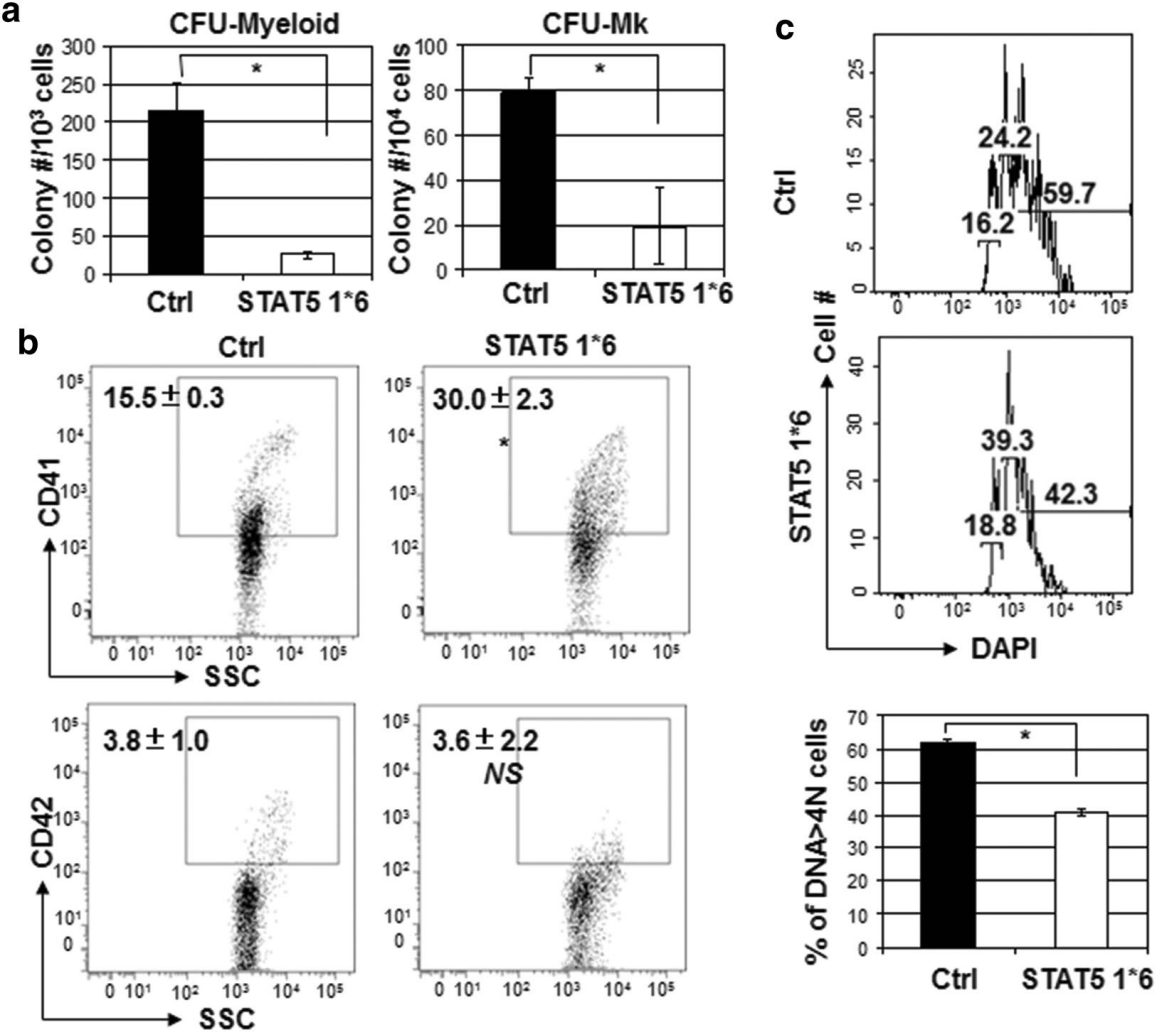
Ectopic expression of constitutively active STAT5 fails to expand CFU-Mk. **a** WT lineage-depleted cells transduced with control retrovirus (Ctrl) or STAT5A1*6-expressing retrovirus (STAT5A1*6) were purified by cell sorting GFP^+^ cells and used for CFU-myeloid and CFU-Mk assay. **b** The transduced cells were also cultured for megakaryocyte differentiation to detect CD41 and CD42 expression. *Numbers* are statistics of two experiments with duplicate and indicate the percentage of the gated cells. **c** The resultant cells were also stained with CD41 and DAPI. A gate was set to analyze the DNA content of CD41^+^ cells by flow cytometry. Numbers indicate the percentage of the gated cells with 2N, 4N or >4N DNA content (from left to right). The bar graph is the statistics of the percentage of polyploid cells (DNA > 4N) from two experiments. *Asterisk* indicates significance (*p* < *0.05*) and *NS* indicates non-significance compared to control

To further test whether STAT5 activation alone is sufficient to promote megakaryocyte expansion, we overexpressed constitutively active STAT5 (STAT5 1*6) in WT mouse bone morrow cells. Unexpectedly, we failed to observe expansion of CFU-Mk or CFU-Myeloid (Fig. 4a). However, CD41 expression was significantly increased and the polyploidy in CD41^+^ cells were reduced in megakaryocyte liquid culture (Fig. 4b, c). Considering that STAT5-deficiency impaired CD41 expression, it is possible that excessive STAT5 activation may drive differentiation at the expense of proliferation of the progenitors and STAT5 may play a critical role in progenitor cells commitment to megakaryocytes. A quantitative STAT5 activation is required in megakaryocyte proliferation and differentiation.

We also tested the function of STAT3 in megakaryocyte proliferation and differentiation. STAT3 with tyrosine phosphorylation site deletion lost its function [36] and mouse with tyrosine site deletion (STAT3 F/F) expressed a truncated form of STAT3 (Additional file 1: Figure S3a). Interestingly, loss of STAT3 function appeared to reduce CFU-Myeloid and CFU-Mk (Additional file 1: Figure S3b). However, overexpression of constitutively active STAT3 (STAT3C) did not support CFU-Mk expansion (Additional file 1: Figure S4a). In both cases, CD41/CD42 expression or polyploidy was not altered (Additional file 1: Figures S3c, d, S4b, c). These observations suggest a distinct role of STAT3 in megakaryocyte proliferation and differentiation.

Tyrosine 625 and 630 affect distinct features of MPL W515L-induced MPNs.

**Fig. 5:**
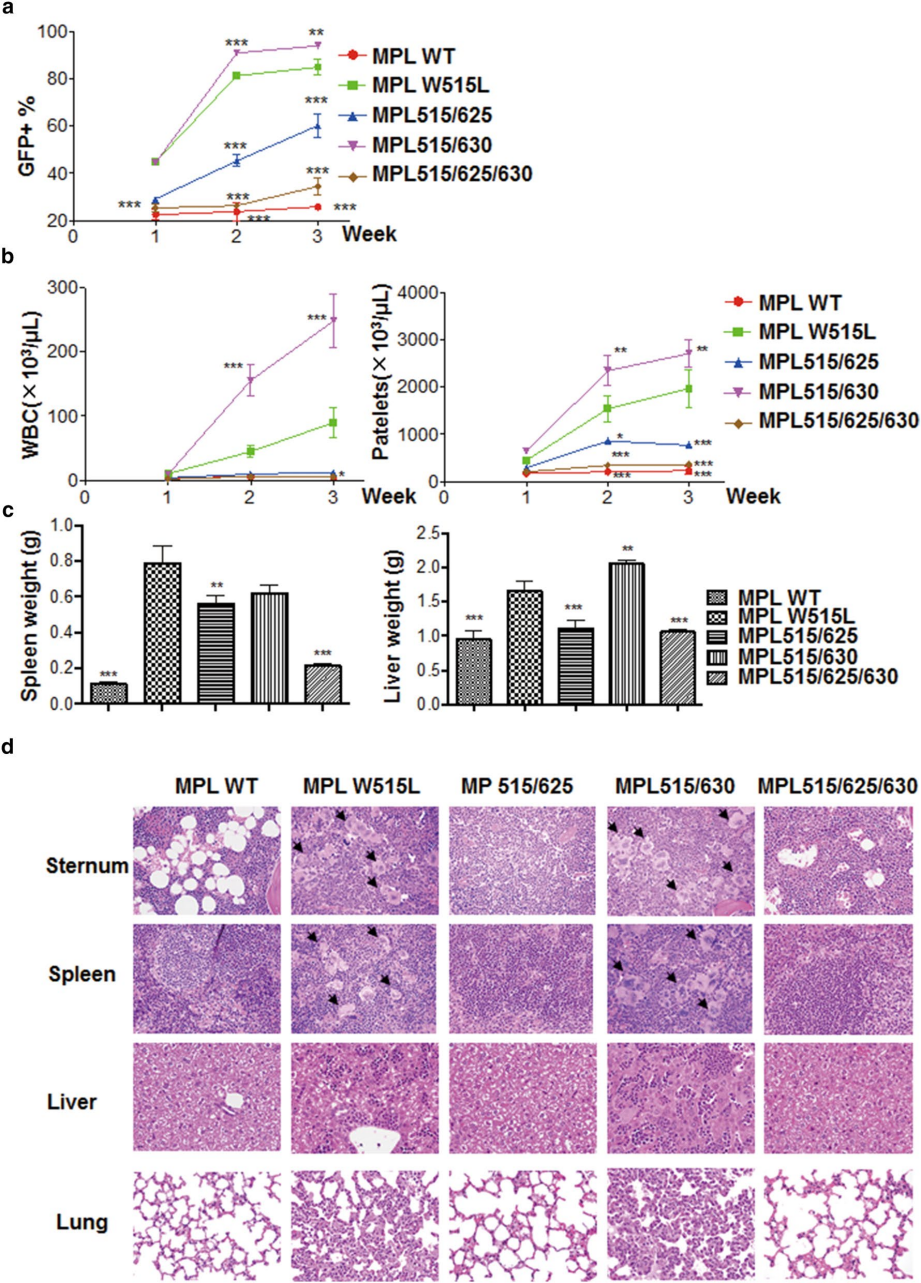
Distinct roles of tyrosine 625 and 630 in MPL W515L-induced MPNs. **a** Mice (*n* = 3) were transplanted with bone marrow progenitor cells transduced with various MPL constructs as indicated. The engraftment of the transduced cells (GFP^+^) in the peripheral blood was monitored by flow cytometry over time as indicated. **b** The white blood counts and the platelet counts in the peripheral blood of the recipient mice were presented. **c** The recipient mice were sacrificed 1 month after transplantation. The spleen and liver weight was measured. **d** Representative photos of the histopathology of sternum, spleen, liver and lung from recipient mice were presented. *Arrows* highlight the megakaryocytes in the H&E sections. *^,^ ** or *** indicates significance (*p* < *0.05*, 0.01, 0.001, respectively) and *NS* indicates non-significance compared to MPL W515L

To examine how tyrosine 625 and 630 affect MPL W515L-induced MPNs in vivo, we performed mouse bone marrow transplantation with all MPL mutants. As shown in Fig. 5a, the GFP^+^ cells were rapidly increased within 3 week in peripheral blood from mice receiving MPL W515L- or MPL515/630-transduced bone marrow cells consistent with their in vitro results (Fig. 1). The GFP^+^ cells for MPL515/630 were even higher than that of MPL W515L. Constant increase of GFP^+^ cells in the peripheral blood from mice receiving MPL515/625-transduced bone marrow cells was also observed (Fig. 5a). In contrast, MPL WT or MPL515/625/630 did not cause significant increase of the GFP^+^ cells. These observations demonstrated that MPL W515L, MPL515/630 and MPL515/625 caused hyperproliferation of bone marrow cells in vivo. Indeed, MPL W515L- or MPL515/630 caused leukocytosis and thrombocytosis (Fig. 5b). MPL515/625 did not cause expansion of white blood cells but did cause mild thrombocytosis (Fig. 5b). Furthermore, MPL W515L, MPL515/625, and MPL515/630 induced splenomegaly, but only MPL W515L and MPL515/630 caused hepatomegaly (Fig. 5c). Pathology analysis of these mice revealed that MPL W515L or MPL515/630 significantly increased megakaryocytes in the bone marrow and spleen and caused blood cell infiltration in liver and lung (Fig. 5d). Of note, MPL515/625 caused hypercellularity in bone marrow similar to MPL W515L and MPL515/630 and MPL515/625/630 did so to a much less extent although MPL515/625 and MPL515/625/630 did not induce typical MPNs (Fig. 5d). These observations suggest that tyrosine 630 may have distinct functions in mediating MPL W515L-induced MPNs.

**Fig. 6:**
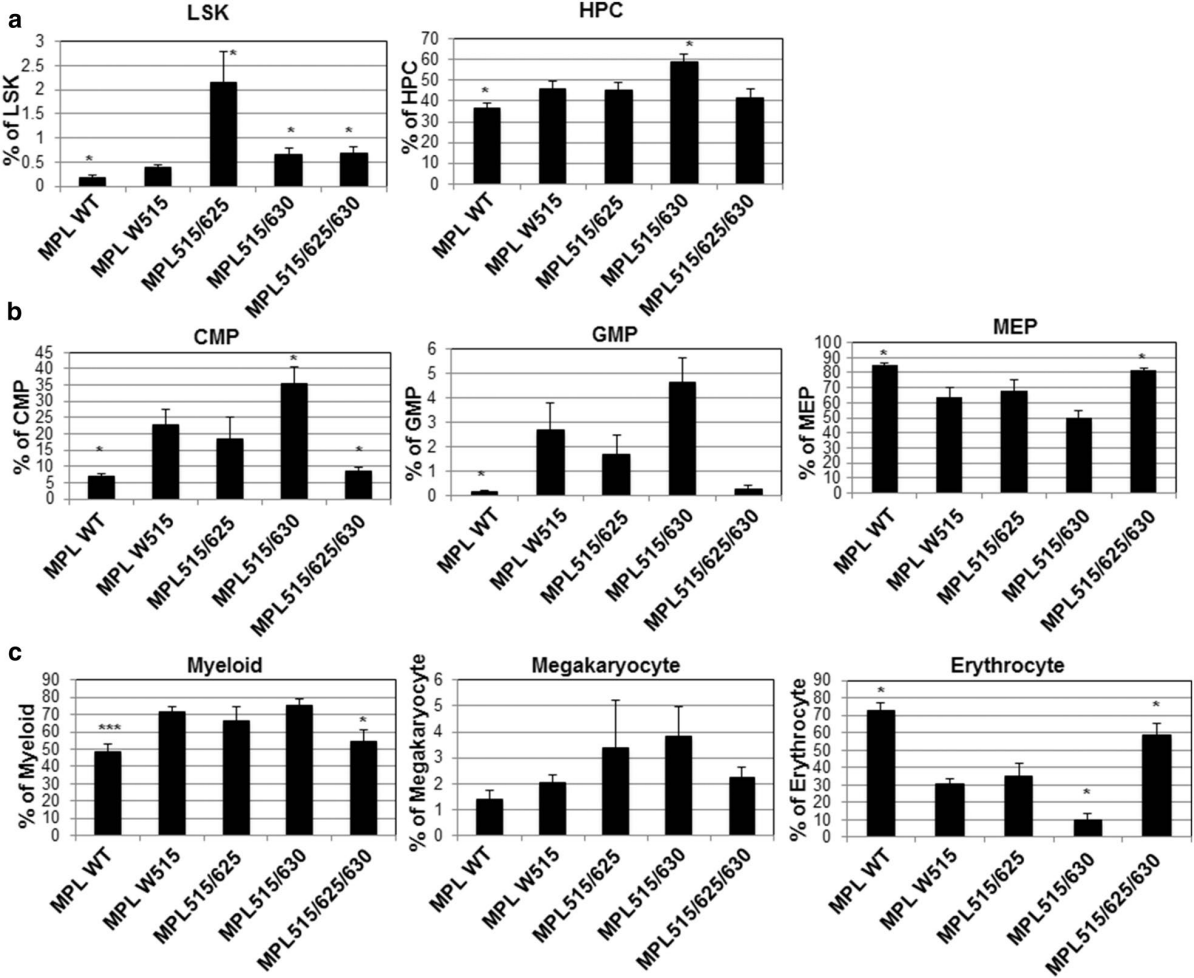
The effect of tyrosine 625 and 630 on hematopoiesis altered by MPL W515L in the bone marrow of recipient mice. **a** Analysis of hematopoietic stem cells (HSC) and progenitor cells (HPC) in the bone marrow cells from mice (*n* = 3) receiving bone marrow cells transduced with different MPL constructs as indicated. **b** Bone marrow cells were also used for analysis of CMP, GMP and MEP. **c** The myeloid, megakaryocyte, or erythrocyte lineages in the bone marrow from recipient mice were also analyzed. *Asterisk* indicates significance (*p* < *0.05*) compared to MPL W515L

A previous report showed that MPL W515L altered the compositions of hematopoietic stem/progenitor cell compartment [37]. To address how tyrosine 625 or 630 affects this, we analyzed the LSK cells (Lin^−^Sca-1^+^c-Kit^+^), progenitor cells (HPC, Lin^−^Sca-1^−^c-Kit^+^), and mature blood cells in the various transplanted animals. As expected, MPL W515L increased LSK and HPC percentage compared to MPL WT in the bone marrow (Fig. 6a; Additional file 1: Figure S5). Interestingly, all other mutant forms of MPL further increased the percentage of LSK with the most significant increase by MPL515/625 although MPL515/625 only caused mild thrombocytosis in peripheral blood (Figs. 5b, 6a; Additional file 1: Figure S5a). In addition, only MPL515/630 further augmented the percentage of HPCs (Fig. 6a; Additional file 1: Figure S5a). Similarly, we observed higher common myeloid progenitor (CMP, CD34^+^FcγR^lo^) and granulocyte-monocyte progenitor (GMP, CD34^+^FcγR^hi^) but not megakaryocyte-erythrocyte progenitor (MEP, CD34^−^FcγR^lo^) percentage in the total progenitor population by MPL W515L compared to MPL WT (Fig. 6b; Additional file 1: Figure S5b). Consistent with a higher disease burden with MPL515/630, more CMP and GMP percentages were observed in MPL 515L/630 compared to that of MPL W515L. Unexpectedly, we observed a decreased percentage of MEP in these mice but not MPL515/625/630 (Fig. 6b). Consistent with the increased percentage of CMP and GMP, we observed an increased percentage of mature myeloid cells (Gr-1^+^Mac-1^+^) in all mutant forms of MPL except MPL515/625/630, possibly at the expense of erythrocytes (Ter119^+^) (Fig. 6c; Additional file 1: Figure S5c). We also observed a trend of increased megakaryocytes in all mutants except MPL515/625/630. Again, these observations demonstrated that tyrosine 630 might play distinct functions in mediating MPL W515L-induced MPNs.

## Discussion

Tyrosine residues 625 and 630 were shown to be critical for normal MPL to induce TPO-dependent cell proliferation in BaF3 cells [25]. In that study, wild-type MPL tyrosine phosphorylation only occurred at these two sites. In addition, STAT3 phosphorylation absolutely required both tyrosine residues whereas STAT5 phosphorylation could be observed in truncated MPL without these two residues. In our study, we demonstrated that MPL W515L induced very different signaling in contrast to normal MPL signaling. For instance, MPL515/625/630 that lost both docking sites for STAT3 still retained the ability to phosphorylated STAT3 in the absence of TPO while wild-type MPL completely failed to do so (Fig. 1d). In contrast, STAT5 was almost completely abrogated without these two tyrosine residues. These observations suggest that MPL W515L may rewire the downstream JAK/STAT signaling pathway and create novel STAT3 docking sites, possibly by phosphorylating other potential tyrosine residues.

Our study also reveals that the MPL W515L-induced TPO-independent cell proliferation may be the converging effect of multiple downstream signaling pathways. In our experiment system, STAT5 and was found to be critical. Instead, STAT3 or RAS/MAPK/ERK was dispensable. To further address the importance of each signaling pathway in MPLW515L-induced TPO-independent cell proliferation, we overexpressed dominant negative forms of STAT3/5 and AKT in MPL W515L-transduced cells and cultured them with or without TPO. We did not observe significant change for the percentage of GFP^+^ cells (Additional file 1: Figure S6). These observations support the idea that single inhibitor targeting specific signaling pathway may not enough to suppressed hyperproliferation induced by MPL W515L. These results also rationalize the combinatory application of inhibitors in MPNs therapy as proposed previously [21, 38, 39]. In addition, we noticed that both STAT5 deficiency and overexpression impaired CFU-Mk (Figs. 3b, 4a) indicating a quantitative requirement of STAT5 activation in progenitor/stem cells. STAT5 activation may balance cell proliferation and differentiation. Indeed, self-renewal and differentiation of hematopoietic stem cells were impaired if the balance of STAT5 activity was broken [40]. This may be a common nature of those critical hematopoiesis regulators including *Gata2* gene since both GATA2 deficiency and overexpression impaired HSC function and caused pancytopenia in mice [41, 42].

Of note, our study further suggests that MPL mutations may also affect HSC and progenitor function and STAT3 might mediate such effect, though it is not required for MPL W515L-induced hyperproliferation. TPO plays an important role in HSC and MPL deficiency showed impaired HSC function [43]. In our system, MPL515/625 only caused splenomegaly and mild thrombocytosis without leukocytosis. However, it did cause hypercellularity and dramatically increased HSC percentage in the bone marrow. In addition, MPL515/625/630 retained the ability to increase HSC percentage with much less hypercellularity in the bone marrow. The apparent common feature in MPL515/625 and MPL515/625/630 was the remaining STAT3 phosphorylation. STAT3 has been shown to play a critical role in stem cell renewal as well as cytokine signaling [44, 45]. However, its role in HSC function has not been fully addressed. Considering that megakaryocytes synthesize many cytokines that influence the microenvironment of the bone marrow for normal hematopoiesis and HSC function, it is possible that the remaining STAT3 phosphorylation somehow affect cytokine production leading to hypercellularity and HSC expansion in the bone marrow. It would be worth of studying the function of MPL W515L on the STAT3-null background.

We also noticed that MPL515/630 caused more severe phenotype than MPL W515L in vivo while MPL515/630 stimulated less proliferation in G1ME cells and comparable CFU-Mk compared to that of MPL W515L in vitro. We speculated that this might be due to the artificial effect of the in vitro system, which removed all cytokines including TPO. It has been reported that some proinflammatory cytokines can be elevated in MPNs patients, which also stimulate JAK/STATs pathways [46]. Another possibility is that inhibitors like phosphatases may compete with STATs to bind tyrosine 630 [24, 47]. Even if loss of tyrosine 630 did not significantly impaired signaling in vitro, it may shift the effect to a worse phenotype due to these combinatory factors in vivo. Thus it is interesting to explore new players that may bind to tyrosine 630 or 625 and be potential targets for therapy.

## Conclusion

Our study revealed distinct features of tyrosine sites 625 and 630 in mediating MPL W515L-induced signaling, megakaryocyte hyperproliferation, and MPNs. Our study also provided experimental evidence showing that MPL cytosolic phosphorylated Y625 and flanking sequences could be potential targets for pharmacologic interference in MPNs.

## Additional file

10.1186/s13578-016-0097-3 **Figure S1.** MPL W515L induces megakaryocyte hyperproliferation in vitro and causes myeloproliferative neoplasm in vivo. **Figure S2.** Cell proliferation and signaling in G1ME cells transduced with various MPL mutants in the presence of TPO. **Figure S3.** The effect of STAT3-deficiency on megakaryopoiesis. **Figure S4.** The effect of STAT3 ectopic expression on the megakaryopoiesis. **Figure S5.** Flow cytometry analysis of BM cells from the recipient mice. **Figure S6.** Inactivating specific signaling pathway was not enough to suppress hyperproliferation induced by MPL W515L.

## Abbreviations

MPN: myeloproliferative neoplasms
CFU-Mk: colony-forming unit megakaryocytes
JAK2: janus kinase 2
STAT5: signal transducer and activator of transcription 5
STAT3: signal transducer and activator of transcription 3
MAPK: mitogen-activated protein kinases
ERK: extracellular signal-regulated kinases
JNK: c-Jun N-terminal kinases
TPO: thrombopoietin
HSC70: heat shock cognate protein 70
PV: polycythemia vera
ET: essential thrombocythemia
PMF: primary myelofibrosis
SCF: stem cell factor
DAPI: 4′, 6-diamidino-2-phenylindole
HSC: hematopoietic stem cell
HPC: hematopoietic progenitor cells
GMP: granulocyte-macrophage progenitor
CMP: common myeloid progenitor
MEP: megakaryocyte-erythroid progenitor

## References

[1] Kralovics R, Passamonti F, Buser AS, Teo SS, Tiedt R, Passweg JR (2005). A gain-of-function mutation of JAK2 in myeloproliferative disorders. N Engl J Med.

[2] Levine RL, Wadleigh M, Cools J, Ebert BL, Wernig G, Huntly BJ (2005). Activating mutation in the tyrosine kinase JAK2 in polycythemia vera, essential thrombocythemia, and myeloid metaplasia with myelofibrosis. Cancer Cell.

[3] Pikman Y, Lee BH, Mercher T, McDowell E, Ebert BL, Gozo M (2006). MPLW515L is a novel somatic activating mutation in myelofibrosis with myeloid metaplasia. PLoS Med.

[4] Li J, Kent DG, Chen E, Green AR (2011). Mouse models of myeloproliferative neoplasms: JAK of all grades. Dis Model Mech.

[5] Mercher T, Raffel GD, Moore SA, Cornejo MG, Baudry-Bluteau D, Cagnard N (2009). The OTT-MAL fusion oncogene activates RBPJ-mediated transcription and induces acute megakaryoblastic leukemia in a knockin mouse model. J Clin Invest.

[6] James C, Ugo V, Le Couedic JP, Staerk J, Delhommeau F, Lacout C (2005). A unique clonal JAK2 mutation leading to constitutive signalling causes polycythaemia vera. Nature.

[7] Wernig G, Mercher T, Okabe R, Levine RL, Lee BH, Gilliland DG (2006). Expression of Jak2V617F causes a polycythemia vera-like disease with associated myelofibrosis in a murine bone marrow transplant model. Blood.

[8] Pardanani A, Tefferi A (2011). Targeting myeloproliferative neoplasms with JAK inhibitors. Curr Opin Hematol.

[9] Vaddi K, Sarlis NJ, Gupta V (2012). Ruxolitinib, an oral JAK1 and JAK2 inhibitor, in myelofibrosis. Expert Opin Pharmacother.

[10] Rampal R, Al-Shahrour F, Abdel-Wahab O, Patel JP, Brunel JP, Mermel CH (2014). Integrated genomic analysis illustrates the central role of JAK-STAT pathway activation in myeloproliferative neoplasm pathogenesis. Blood.

[11] Walz C, Ahmed W, Lazarides K, Betancur M, Patel N, Hennighausen L (2012). Essential role for Stat5a/b in myeloproliferative neoplasms induced by BCR-ABL1 and JAK2(V617F) in mice. Blood.

[12] Sillaber C, Gesbert F, Frank DA, Sattler M, Griffin JD (2000). STAT5 activation contributes to growth and viability in Bcr/Abl-transformed cells. Blood.

[13] de Groot RP, Raaijmakers JA, Lammers JW, Jove R, Koenderman L (1999). STAT5 activation by BCR-Abl contributes to transformation of K562 leukemia cells. Blood.

[14] Gough DJ, Marie IJ, Lobry C, Aifantis I, Levy DE (2014). STAT3 supports experimental K-RasG12D-induced murine myeloproliferative neoplasms dependent on serine phosphorylation. Blood.

[15] Velho S, Oliveira C, Ferreira A, Ferreira AC, Suriano G, Schwartz S (2005). The prevalence of PIK3CA mutations in gastric and colon cancer. Eur J Cancer.

[16] Li VS, Wong CW, Chan TL, Chan AS, Zhao W, Chu KM (2005). Mutations of PIK3CA in gastric adenocarcinoma. BMC Cancer.

[17] Samuels Y, Wang Z, Bardelli A, Silliman N, Ptak J, Szabo S (2004). High frequency of mutations of the PIK3CA gene in human cancers. Science.

[18] Kharas MG, Okabe R, Ganis JJ, Gozo M, Khandan T, Paktinat M (2010). Constitutively active AKT depletes hematopoietic stem cells and induces leukemia in mice. Blood.

[19] Khan I, Huang Z, Wen Q, Stankiewicz MJ, Gilles L, Goldenson B (2013). AKT is a therapeutic target in myeloproliferative neoplasms. Leukemia.

[20] Fiskus W, Verstovsek S, Manshouri T, Smith JE, Peth K, Abhyankar S (2013). Dual PI3K/AKT/mTOR inhibitor BEZ235 synergistically enhances the activity of JAK2 inhibitor against cultured and primary human myeloproliferative neoplasm cells. Mol Cancer Ther.

[21] Bartalucci N, Tozzi L, Bogani C, Martinelli S, Rotunno G, Villeval JL (2013). Co-targeting the PI3K/mTOR and JAK2 signalling pathways produces synergistic activity against myeloproliferative neoplasms. J Cell Mol Med.

[22] Staser K, Park SJ, Rhodes SD, Zeng Y, He YZ, Shew MA (2013). Normal hematopoiesis and neurofibromin-deficient myeloproliferative disease require Erk. J Clin Invest.

[23] Chung E, Hsu CL, Kondo M (2011). Constitutive MAP kinase activation in hematopoietic stem cells induces a myeloproliferative disorder. PLoS One.

[24] Sangkhae V, Saur SJ, Kaushansky A, Kaushansky K, Hitchcock IS (2014). Phosphorylated c-Mpl tyrosine 591 regulates thrombopoietin-induced signaling. Exp Hematol.

[25] Drachman JG, Kaushansky K (1997). Dissecting the thrombopoietin receptor: functional elements of the Mpl cytoplasmic domain. Proc Natl Acad Sci USA.

[26] Huang Z, Richmond TD, Muntean AG, Barber DL, Weiss MJ, Crispino JD (2007). STAT1 promotes megakaryopoiesis downstream of GATA-1 in mice. J Clin Invest.

[27] Huang Z, Dore LC, Li Z, Orkin SH, Feng G, Lin S (2009). GATA-2 reinforces megakaryocyte development in the absence of GATA-1. Mol Cell Biol.

[28] Wen Q, Goldenson B, Silver SJ, Schenone M, Dancik V, Huang Z (2012). Identification of regulators of polyploidization presents therapeutic targets for treatment of AMKL. Cell.

[29] Alexander WS, Maurer AB, Novak U, Harrison-Smith M (1996). Tyrosine-599 of the c-Mpl receptor is required for Shc phosphorylation and the induction of cellular differentiation. EMBO J.

[30] Jiang F, Jia YZ, Cohen I (2002). Fibronectin- and protein kinase C-mediated activation of ERK/MAPK are essential for proplateletlike formation. Blood.

[31] Minamiguchi H, Kimura T, Urata Y, Miyazaki H, Bamba T, Abe T (2001). Simultaneous signalling through c-mpl, c-kit and CXCR4 enhances the proliferation and differentiation of human megakaryocyte progenitors: possible roles of the PI3-K, PKC and MAPK pathways. Br J Haematol.

[32] Fiskus W, Verstovsek S, Manshouri T, Smith JE, Peth K, Abhyankar S (2013). Dual PI3K/AKT/mTOR inhibitor BEZ235 synergistically enhances the activity of JAK2 inhibitor against cultured and primary human myeloproliferative neoplasm cells. Mol Cancer Ther.

[33] Lee MS, Igawa T, Lin MF (2004). Tyrosine-317 of p52(Shc) mediates androgen-stimulated proliferation signals in human prostate cancer cells. Oncogene.

[34] Ishihara H, Sasaoka T, Wada T, Ishiki M, Haruta T, Usui I (1998). Relative involvement of Shc tyrosine 239/240 and tyrosine 317 on insulin induced mitogenic signaling in rat1 fibroblasts expressing insulin receptors. Biochem Biophys Res Commun.

[35] Ye D, Wolff N, Li L, Zhang S, Ilaria RL (2006). STAT5 signaling is required for the efficient induction and maintenance of CML in mice. Blood.

[36] Kirito K, Osawa M, Morita H, Shimizu R, Yamamoto M, Oda A (2002). A functional role of Stat3 in in vivo megakaryopoiesis. Blood.

[37] Chaligne R, James C, Tonetti C, Besancenot R, Le Couedic JP, Fava F (2007). Evidence for MPL W515L/K mutations in hematopoietic stem cells in primitive myelofibrosis. Blood.

[38] Choong ML, Pecquet C, Pendharkar V, Diaconu CC, Yong JW, Tai SJ (2013). Combination treatment for myeloproliferative neoplasms using JAK and pan-class I PI3K inhibitors. J Cell Mol Med.

[39] Wang Y, Fiskus W, Chong DG, Buckley KM, Natarajan K, Rao R (2009). Cotreatment with panobinostat and JAK2 inhibitor TG101209 attenuates JAK2V617F levels and signaling and exerts synergistic cytotoxic effects against human myeloproliferative neoplastic cells. Blood.

[40] Schepers H, Wierenga AT, Vellenga E, Schuringa JJ (2012). STAT5-mediated self-renewal of normal hematopoietic and leukemic stem cells. JakStat.

[41] Persons DA, Allay JA, Allay ER, Ashmun RA, Orlic D, Jane SM (1999). Enforced expression of the GATA-2 transcription factor blocks normal hematopoiesis. Blood.

[42] Tsai FY, Keller G, Kuo FC, Weiss M, Chen J, Rosenblatt M (1994). An early haematopoietic defect in mice lacking the transcription factor GATA-2. Nature.

[43] Abkowitz JL, Chen J (2007). Studies of c-Mpl function distinguish the replication of hematopoietic stem cells from the expansion of differentiating clones. Blood.

[44] Niwa H, Burdon T, Chambers I, Smith A (1998). Self-renewal of pluripotent embryonic stem cells is mediated via activation of STAT3. Genes Dev.

[45] Kristensen DM, Kalisz M, Nielsen JH (2005). Cytokine signalling in embryonic stem cells. APMIS.

[46] Cokic VP, Mitrovic-Ajtic O, Beleslin-Cokic BB, Markovic D, Buac M, Diklic M (2015). Proinflammatory cytokine IL-6 and JAK-STAT signaling pathway in myeloproliferative neoplasms. Mediators Inflamm.

[47] Pozner R, Schattner M (2008). TPO signaling: when the tyrosines go marching in(side). Blood.

