# Supplementary material for: Tyrosine 625 plays a key role and cooperates with tyrosine 630 in MPL W515L-induced signaling and myeloproliferative neoplasms
Source: Cell Biosci. 2016 May 23;6:34. doi: 10.1186/s13578-016-0097-3 (PMC4877759; doi:10.1186/s13578-016-0097-3)
Supplement: Supplementary file 1 — 10.1186/s13578-016-0097-3 Figure S1. MPL W515L induces megakaryocyte hyperproliferation in vitro and causes myeloproliferative neoplasm in vivo. Figure S2. Cell proliferation and signaling in G1ME cells transduced with various MPL mutants in the presence of TPO. Figure S3. The effect of STAT3-deficiency on megakaryopoiesis. Figure S4. The effect of STAT3 ectopic expression on the megakaryopoiesis. Figure S5. Flow cytometry analysis of BM cells from the recipient mice. Figure S6. Inactivating specific signaling pathway was not enough to suppress hyperproliferation induced by MPL W515L. [file 13578_2016_97_MOESM1_ESM.docx]

**Supplemental information**

**
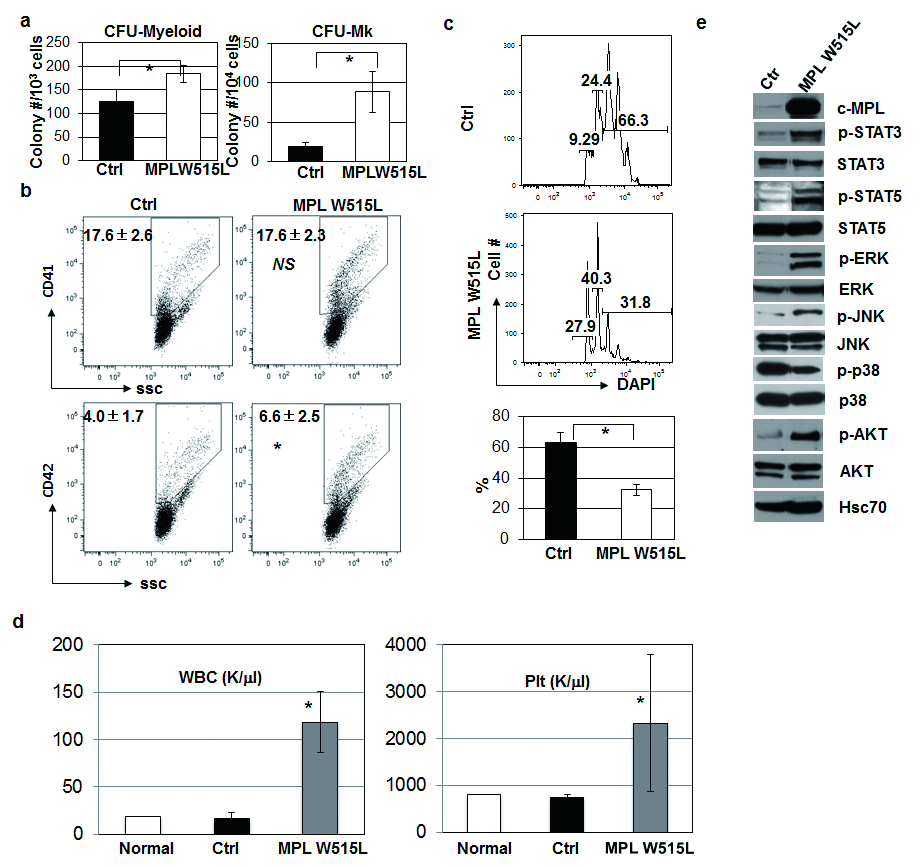
**

**Fig. S1** MPL W515L induces megakaryocyte hyperproliferation in vitro and causes myeloproliferative neoplasm in vivo. **a** WT lineage-depleted bone marrow cells transduced with control retrovirus (Ctrl) or MPL W515L-expressing retrovirus and selected with puromycin were used for colony-forming unit-myeloid (CFU-Myeloid) or megakaryocytes (CFU-Mk). Results were statistics of three independent experiments with duplicates. **b** The transduced cells were also cultured in the megakaryocyte differentiation medium for three days and stained with CD41 and CD42 antibodies. Megakaryocyte differentiation of the resultant cells was analyzed by flow cytometry. Numbers indicate the percentage of the gated cells. Results were statistics of three independent experiments with duplicates. Representative dot plots were presented. **c** The resultant cells were also stained with CD41 antibody and DAPI. A gate was set to analyze the DNA content in the CD41+ cells by flow cytometry. Numbers indicate the percentage of the gated cells. The bar graph is the statistics of the percentage of polyploid cells (DNA>4N) from three independent experiments with duplicates. Representative histograms were presented. **d** The transduced cells were transplanted into syngenic wild-type mice (n=3). The white blood count (WBC) and platelet count in the peripheral blood were detected one month after transplantation. Normal indicates mice without irradiation and transplantation. **e** Cells were collected from CFU-Myeloid on day 7 and used for western blot to detect phosphorylation or total level of proteins as indicated. * indicates significance (p<0.05) and NS indicates non-significance compare to control.

**
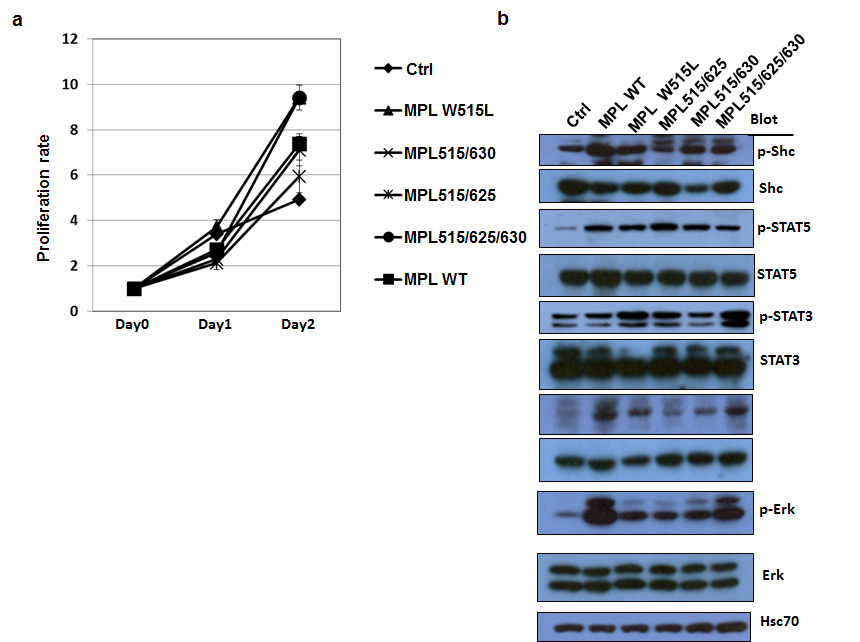
**

**Fig. S2** Cell proliferation and signaling in G1ME cells transduced with various MPL mutants in the presence of TPO. **a** G1ME cells transduced with each construct were cultured in the presence of TPO for 2 days. The cell numbers on each day were counted under microscopy and normalized to the starting cell numbers (Day 0) and presented as proliferation rate. Results were statistics of a representative experiment with duplicates from two independent experiments with similar results. **b** G1ME cells transduced with each construct were cultured in the presence of TPO. Cells were harvested for western blot to detect the phosphorylation and the expression level of each protein as indicated. Hsc70 serves as loading control.


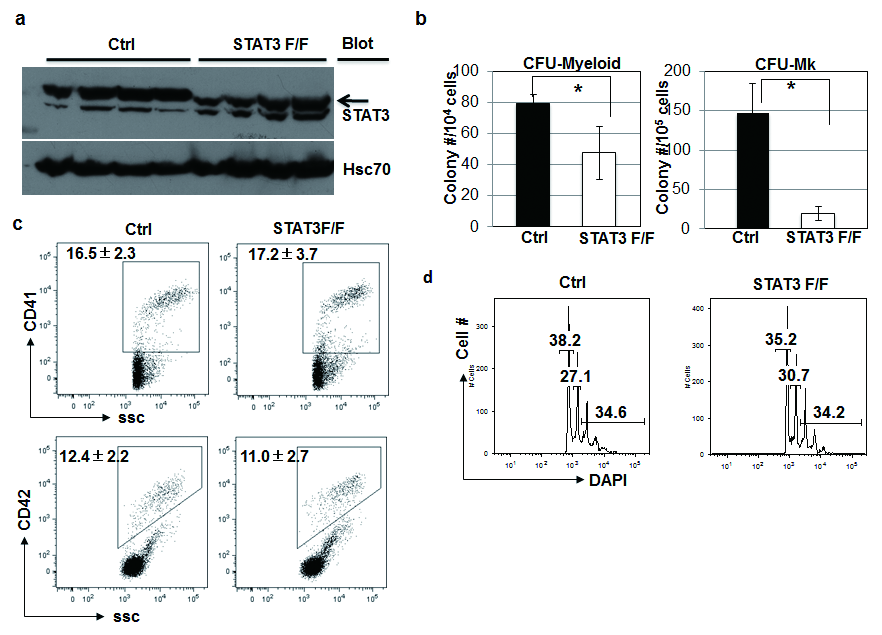


**Fig. S3** The effect of STAT3-deficiency on megakaryopoiesis. **a** Control and STAT3F/F mice were treated with pI-pC and the excision of STAT3 in the bone marrow cells were confirmed by western blot to detect the protein level of full length and truncated STAT3. Arrow indicates the production of truncated STAT3 protein after excision. **b** The control (Ctrl) or STAT3-deficient (STAT3 F/F) bone marrow cells were used for colony-forming unit-myeloid cells (CFU-Myeloid) and megakaryocytes (CFU-Mk). **c** The cells were also cultured in the differentiation medium with TPO. The megakaryocyte differentiation was measured by detecting the expression of megakaryocyte specific markers CD41 and CD42 with flow cytometry. **d** The resultant cells were stained for CD41 with fluorescence-labeled anti-CD41 antibody and stained for DNA with DAPI. The DNA content in the CD41+ cells was analyzed by flow cytometry. Cells with DNA content > 8N were considered as polyploid cells.


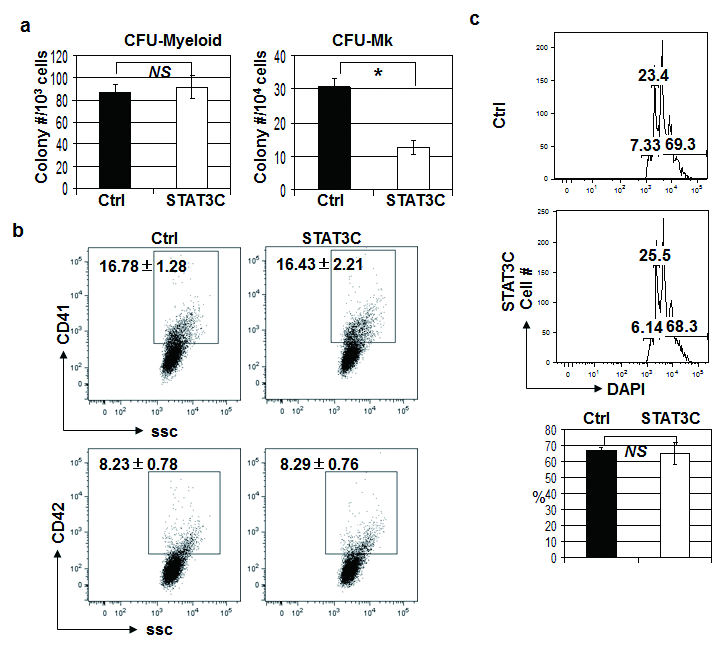


**Fig. S4** The effect of STAT3 ectopic expression on the megakaryopoiesis. **a** Bone marrow progenitor cells were transduced with control retrovirus (Ctrl) or retrovirus expressing constitutively active form of STAT3 (STAT3C). The transduced cells were selected with puromycin and used for colony-forming unit-myeloid cells (CFU-Myeloid) or megakaryocyte (CFU-Mk). **b** The transduced cells were cultured in the presence of differentiation medium with TPO for 3 days. The megakaryocyte differentiation was measured by detecting the expression of megakaryocyte specific markers CD41 and CD42 and analyzed with flow cytometry. **c** The resultant cells were stained for CD41 with fluorescence-labeled anti-CD41 antibody and stained for DNA with DAPI. The DNA content in the CD41+ cells was analyzed by flow cytometry. Cells with DNA content > 8N were considered as polyploid cells.


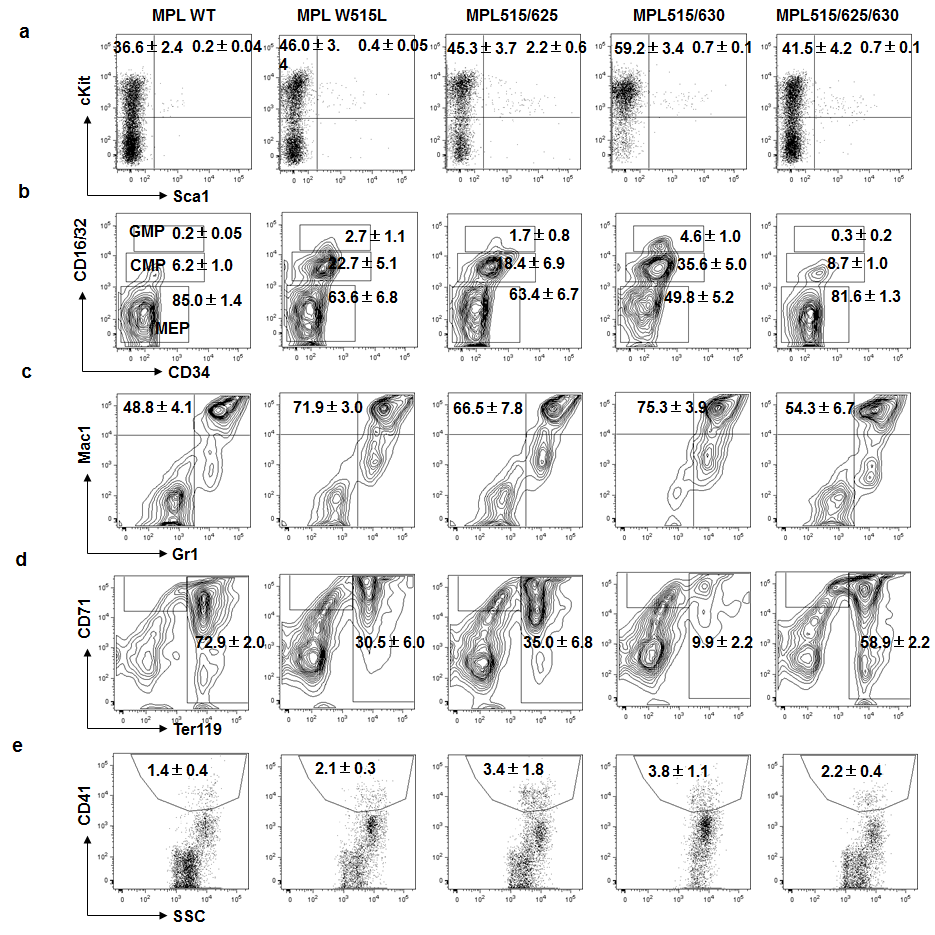


**Fig. S5** Flow cytometry analysis of BM cells from the recipient mice. Mice were transplanted with bone marrow cells transduced with various MPL constructs as indicated. The bone marrow cells from the recipient mice were used for flow cytometry analysis and a GFP+ gate was set for further analysis. **a** Representative dot plots of flow cytometry analysis for LSK cells and HPC cells in lineage negative bone marrow cells from recipient mice. LSK and HPC were defined as Lin^-^Sca1^+^c-kit^+^ and Lin^-^Sca1^-^c-kit^+^ respectively. **b** Representative plots of flow cytometry analysis for CMP, GMP and MEP populations in HPCs defined as CD34^+^FcγR^lo^, CD34^+^FcγR^hi^, and CD34^-^FcγR^lo^, respectively. **c** Representative plots of flow cytometry analysis for myeloid cells with the percentage of Mac1+/Gr1+ cells. **d** Representative plots of flow cytometry analysis for Ter119+ erythrocytes. **e** Representative plots of flow cytometry analysis for as CD41+ megakaryocytes in the bone marrow.

**
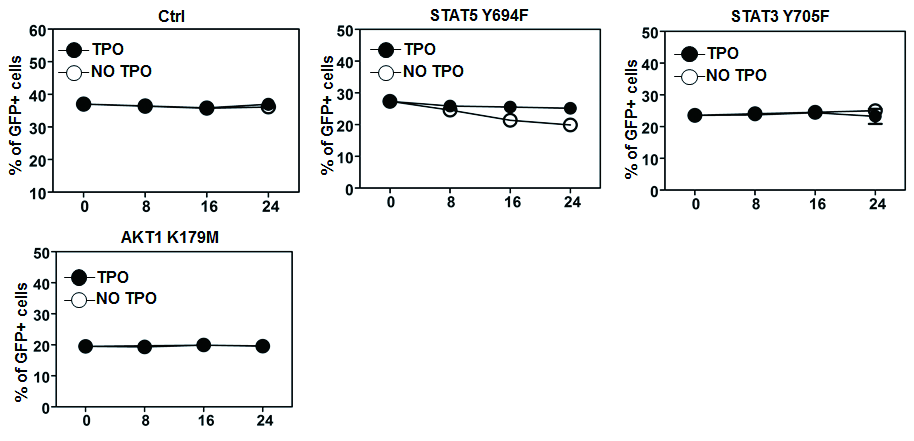
**

**Fig. S6** The effect of downstream signaling molecules on MPL W515L-induced cell proliferation or survival. MPL W515L-transduced G1ME cells were infected with control virus expressing GFP or virus bicistronically expressing GFP and dominant negative form of signaling molecules as indicated. The resultant cells were further cultured with (solid round) or without TPO (hollow round). The percentages of GFP+ in the resultant cells at different time points post infection were analyzed by flow cytometry.
